# Supplementary material for: Context-invariant beliefs are supported by dynamic reconfiguration of single unit functional connectivity in prefrontal cortex of male macaques
Source: Nat Commun. 2024 Jul 9;15:5738. doi: 10.1038/s41467-024-50203-5 (PMC11233555; doi:10.1038/s41467-024-50203-5)
Supplement: Supplementary file 1 — Supplementary Information [file 41467_2024_50203_MOESM1_ESM.pdf]

Supplementary Materials for:

Context-invariant beliefs are supported by dynamic reconfiguration of single unit functional connectivity in prefrontal cortex of male macaques

Jean-Paul Noel[1, *], Edoardo Balzani[1, *], Cristina Savin[1, #], Dora E. Angelaki[1, #]

[1] Center for Neural Science, New York University, New York City, NY, USA
* These authors contributed equally
# These authors jointly supervised this work

Correspondence:
Dr. Jean-Paul Noel
Jpn5@nyu.edu

Supplementary Figures and Figure Captions

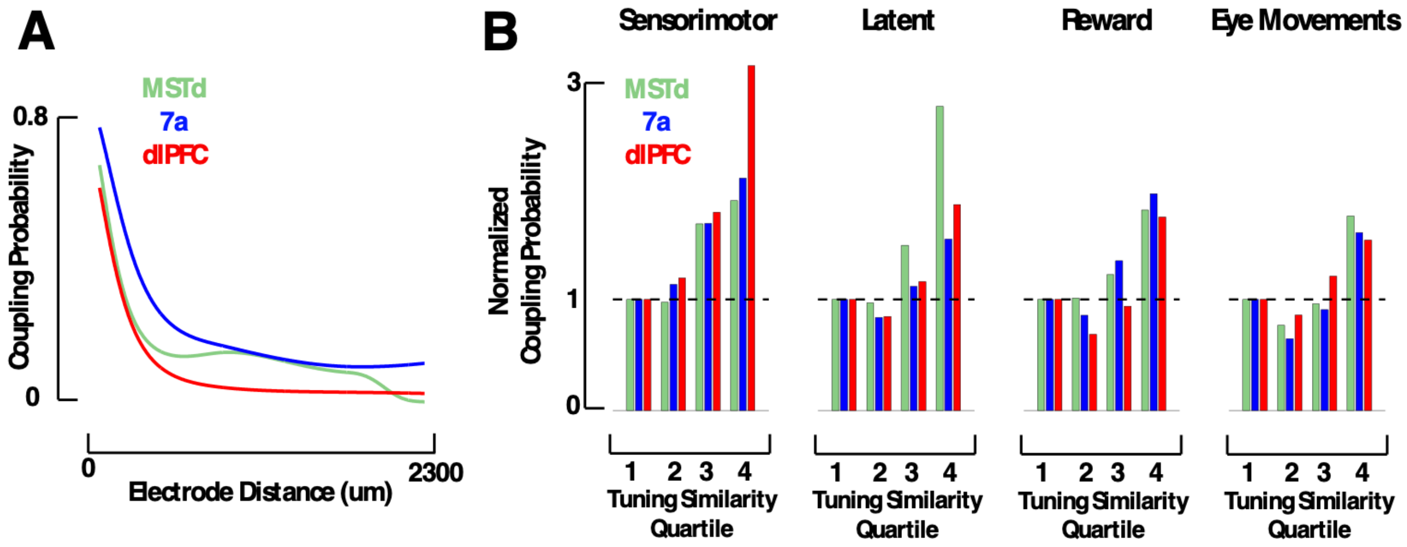

Figure S1. General coupling filter behavior. A. Coupling probability (y-axis) between two neurons as a function of the distance between them (x-axis). This illustration is for data from a single monkey (Monkey S) with recordings in MSTd (green, number of coupling pairs = 1714), area 7a (blue, number of coupling pairs = 97711), and dlPFC (red, number of coupling pairs = 41665). As expected, neurons that are closer to each other are more likely to be coupled. Given this effect and that multiple recording techniques were used (with different spacing between electrodes, 400um in Utah array and 100um in linear probes), we used these estimates to correct coupling probabilities to a single distance (500um). **B.** Coupling probability between any two units given their tuning similarity to sensorimotor, latent, or other (reward and eye positions) variables (data from 3 monkeys). Tuning similarity is computed as the correlation in tuning functions, then these are discretized in quartiles by their tuning similarity (r^2 = [0-.25; 0.25-0.5; 0.5-0.75; 0.75-1]) and averaged within each category (e.g., sensorimotor or latent) and tuning similarity bin. The coupling probability is expressed as a ratio, normalized to the bin with lowest tuning similarity (leftmost), such that a normalized coupling probability of ~3 (e.g., sensorimotor variables in dlPFC) indicates that coupling is three times more likely given high vs. low tuning similarity. Source data are provided as a Source data file.

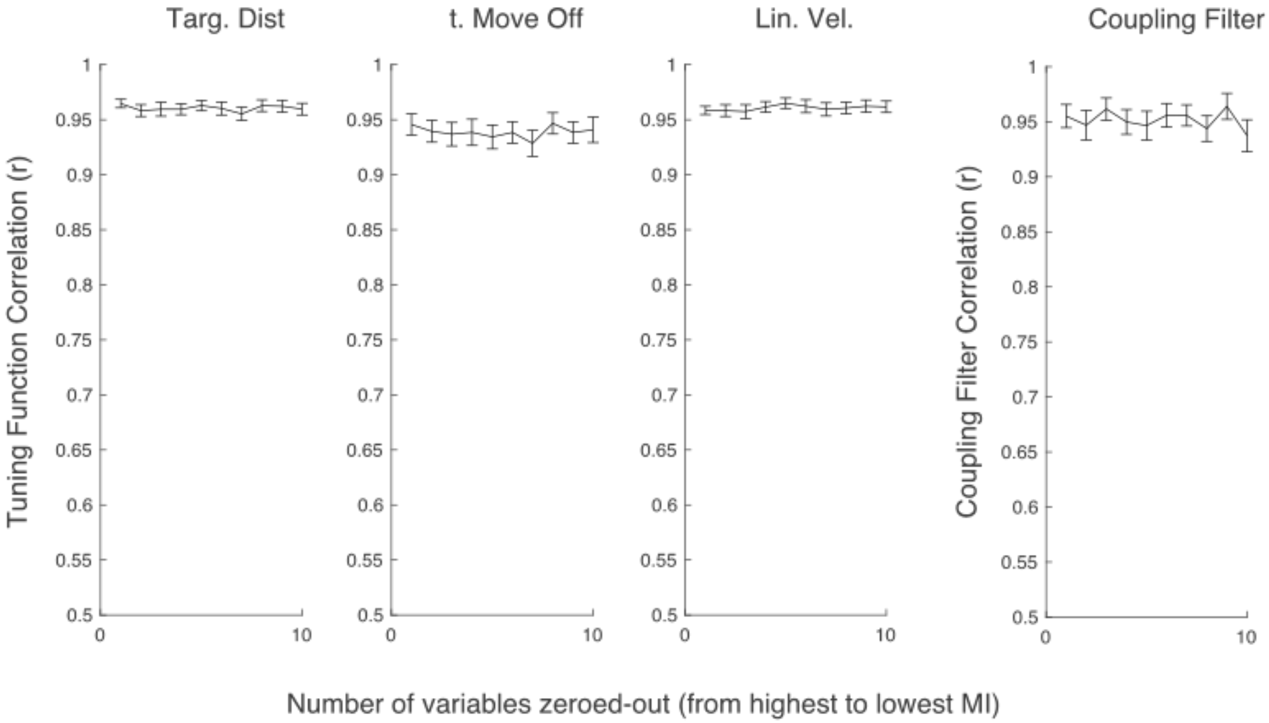

Figure S2. P-GAM validation against ground-truth data. To test for the ability of our encoding model (P-GAM) to recover ground-truth data within the statistical regime of the current dataset, we artificially zeroed out variables that a first full fit deemed to significantly account for variance, and then fit again. More specifically, we sequentially zero out 1 to 10 significant task variables, in order of mutual information (from most to least). On no occasion (0.00%) did a neuron originally deemed to be tuned to either distance to target, movement offset, liner velocity, or the spiking of other neurons (i.e., coupling filters), was it deemed not to be tuned to this variable in re-fits (>20k re-fits). Similarly, on no occasion (0.00%) was a neuron not originally estimated tuned to one of the above-mentioned variables, considered tuned on follow-up fits (see REF[30] for a similar analysis demonstrating no mis-categorizations when recordings are over 20 minutes long. Here they are ~ 180 minutes long). Now, we can be more stringent, and not only examine if neurons change categorization from tuned to not-tuned (or vis-versa) while zeroing out other variables, but we can also examine the shape of the resultant tuning functions. Each time, we examine the correlation between the original and re-fitted tuning functions to target distance (left-most panel), movement offset (second panel), linear velocity (third panel), as well as noise correlations (right-most panel), while zeroing out other variables (e.g., linear acceleration). The tuning and coupling functions remain very stable (~ range = 0.9 to 1.0). Importantly, there is also no evidence for the fact that tuning function correlation becomes unstable after a larger number of variables have been removed (one-way ANOVA, all $p > 0.79$). As such, it appears that the encoding model is appropriately isolating tuning and coupling functions, without parameters standing in for one another. Source data are provided as a Source data file.

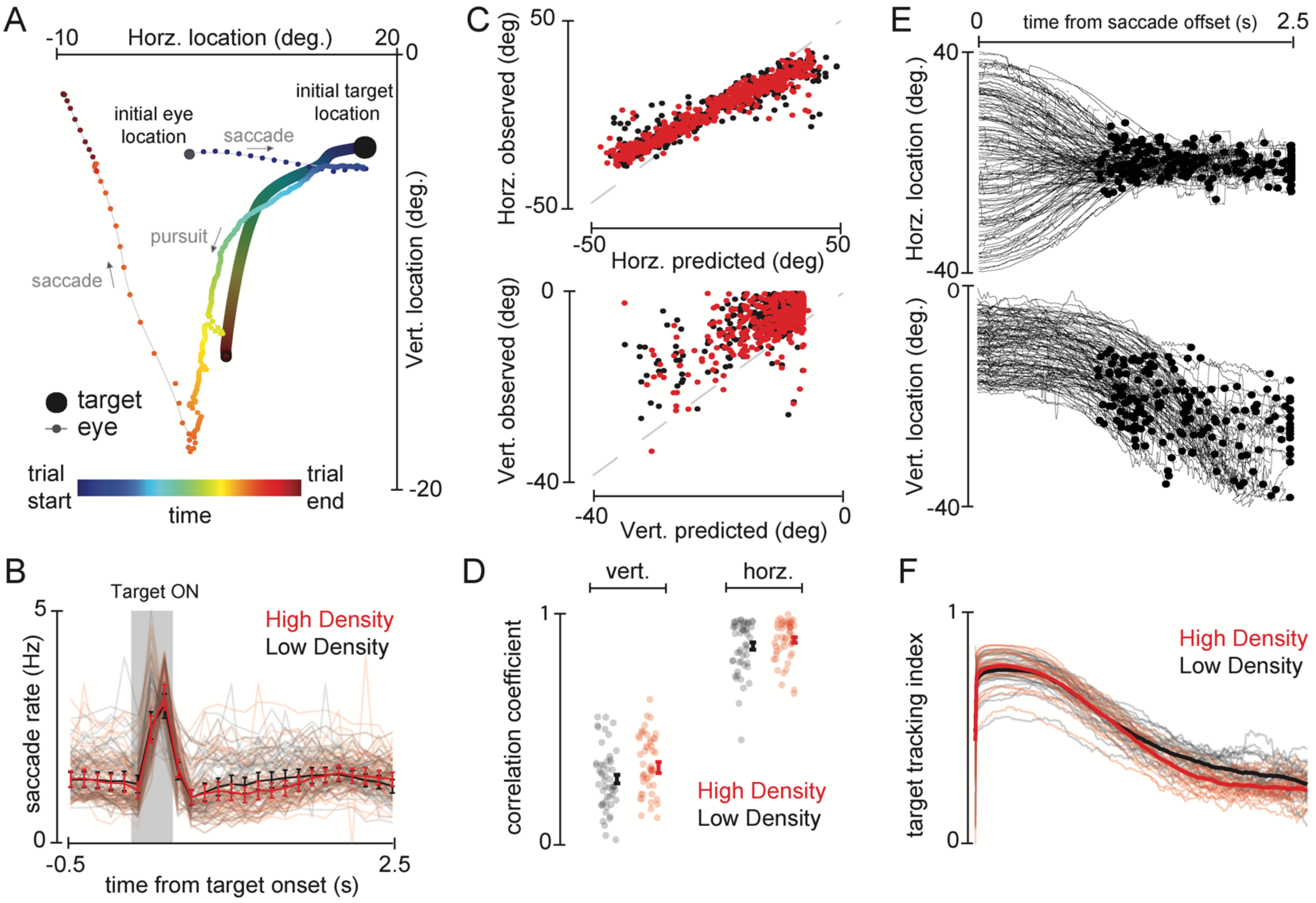

Figure S3. Eye movements during different optic-flow density conditions. **A.** Example trial demonstrating eye movements in screen-coordinates. Upon target presentation, the monkey saccades to this location. Then, as the monkey approaches the target, this latter one progressively becomes more central and shifts downward (even if invisible at this time). The animal's gaze pursuit matches the trajectory of the target. Once the animal stops (response given), the animal saccades to a random location. **B.** Saccade rate (y-axis) as a function of time from target onset (x-axis) and optic-flow density (red = high density; black = low-density). As demonstrated by the example in **A.,** animals increase their likelihood of making saccades briefly after the target is presented. There is no difference between optic flow conditions (t-test at each time bin, all $p > 0.19$, uncorrected). Solid lines are averages across all monkeys; transparent lines are individual sessions. Error bars are ± 1 S.E.M **C.** Correlation between the observed saccade (y-axis, top panel = horizontal axis, bottom panel = vertical axis) and the predicted saccade animals should have made if they were intending to land on the location of the target (x-axis). This is one example session, for both high- (red) and low-density (black) optic flow conditions. Dots are individual trials. **D.** Correlation coefficient (y-axis) for observed and predicted saccades along the vertical and horizontal axis, as a function of optic flow. Dots are individual sessions, error bars show the mean and ± 1 S.E.M. Saccades were better correlated with target location along the horizontal axis ($p < 0.001$), with no difference across optic-flow densities (all $p > 0.39$). **E.** Gaze pursuit for an example session. As shown by the example trial in **A.** horizontal gaze progressively became more central, while vertically the eyes moved downward – as if tracking the invisible target. **F.** We express the correlation between the observed and predicted 2-dimensional gaze location, under the hypothesis that animals are tracking the invisible target (see[25] for the original report of this effect and further methods). We call this the target-tracking index. This index was highest at target offset, and was significantly above chance (y-axis = 0) for the duration of the trials. We observed no difference between high- and low-density optic flow conditions (all $p > 0.61$, time-resolved t-test uncorrected). See REF[25] for the same result in different animals/sessions. Source data are provided as a Source data file.

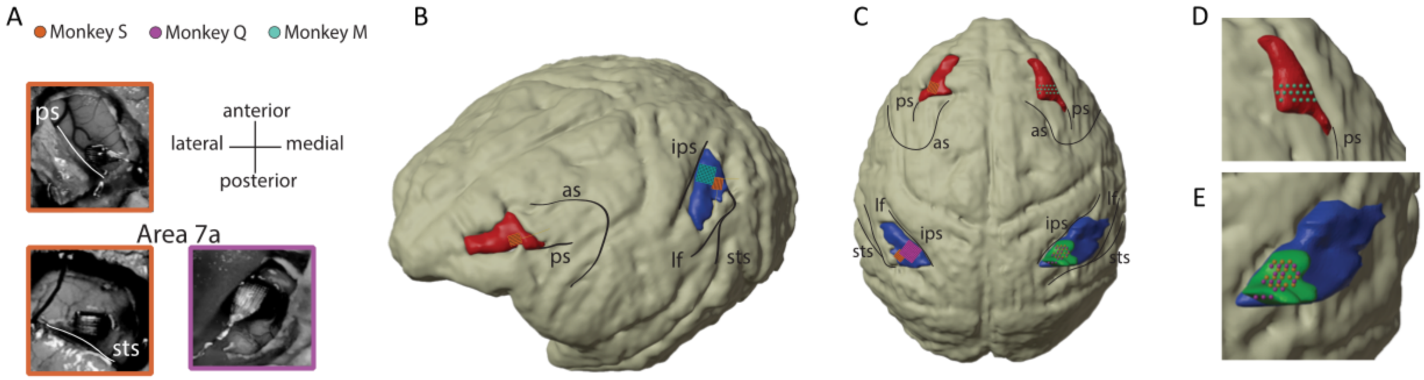

Figure S4. Images and reconstruction of recording sites. A. Pictures showing the location of Utah array implants in Monkey S (orange, top = dorso-lateral pre-frontal cortex, bottom = area 7a) and Monkey Q (area 7a). **B-E.** Magnetic resonance imaging (MRI) reconstruction. The rendering of the brain is from Monkey S, and shows all recording sites (arrays and linear probes) on this common reference. Location of acute recordings with linear probes are indicated by spheres, color coded by monkey (S in orange, Q in purple, and M in cyan). Location of Utah arrays are indicated by squares, also color coded by monkey. Brain areas are also indicated by color (dlPFC in red, area 7a in blue, and MSTd in green). MSTd is directly ventral to 7a and shown on the surface here for illustration). AS, arcuate sulcus; IPS, intraparietal sulcus; PS, principal sulcus; STS, superior temporal sulcus; LF, lateral fissure. Figure adapted from REF[35]. Source data are provided as a Source data file.

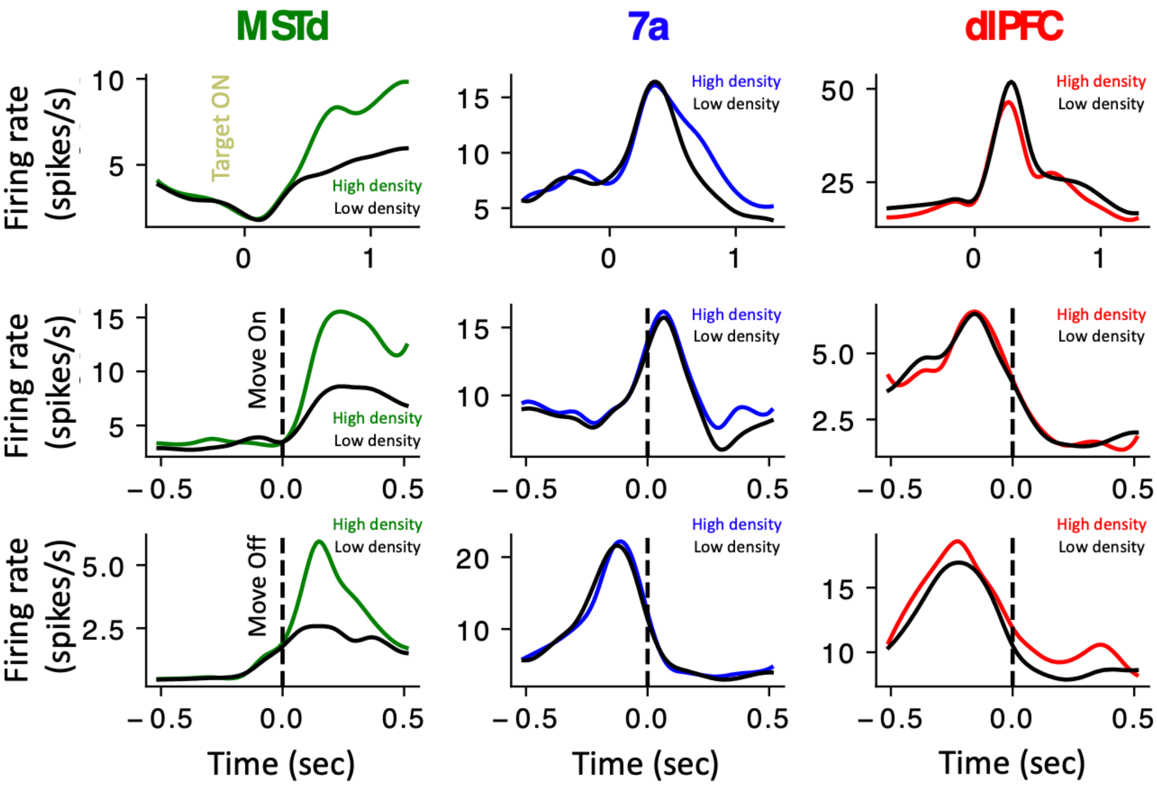

Figure S5. Example peri-event time histograms (PETH). Nine example PETH, three per brain area (MSTd, 7a, and dlPFC, respectively in green, blue, and red) and per event; target onset (top row), macaque movement onset (middle row) and macaque movement offset (bottom row). Most importantly, the PETHs are plotted separately for the high density optic flow condition (colored by brain area) and the low density optic flow conditions (black). The examples are representative (see main text and **Figure 2B**) in that MSTd is gain modulated (gain <1) from the high to low density condition, while responses in 7a and dlPFC were less impacted by the density of optic flow. Source data are provided as a Source data file.

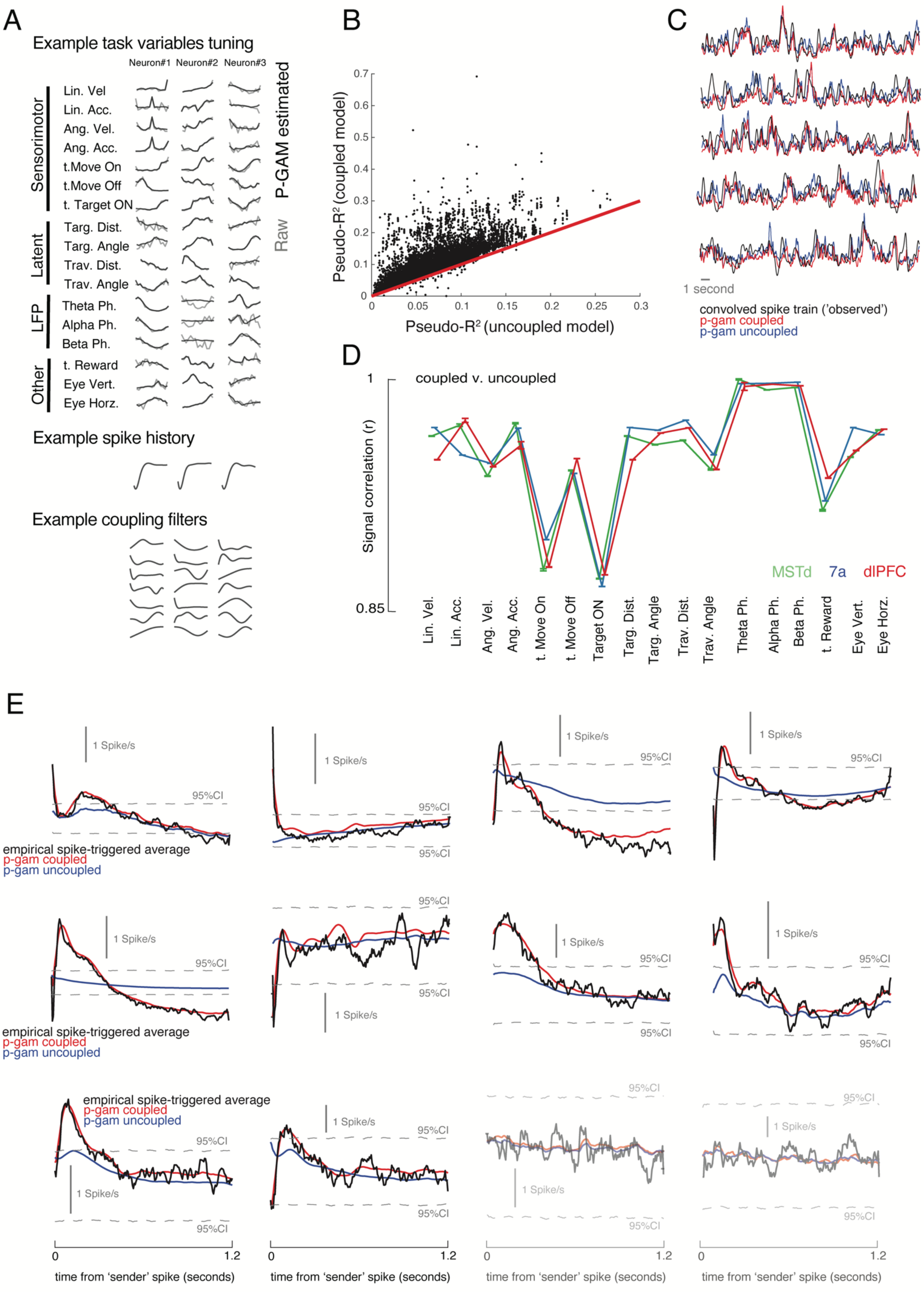

A
Example task variables tuning
Neuron#1 Neuron#2 Neuron#3
Sensorimotor
Lin. Vel
Lin. Acc.
Ang. Vel.
Ang. Acc.
t.Move On
t.Move Off
t. Target ON
Latent
Targ. Dist.
Targ. Angle
Trav. Dist.
Trav. Angle
LFP
Theta Ph.
Alpha Ph.
Beta Ph.
Other
t. Reward
Eye Vert.
Eye Horz.
P-GAM estimated
Raw
Example spike history
Example coupling filters
B
Pseudo-R² (coupled model)
Pseudo-R² (uncoupled model)
C
1 second
convolved spike train ('observed')
p-gam coupled
p-gam uncoupled
D
coupled v. uncoupled
Signal correlation (r)
MSTd 7a dlPFC
Lin. Vel. Lin. Acc. Ang. Vel. Ang. Acc. t. Move On t. Move Off Target ON Targ. Dist. Targ. Angle Trav. Dist. Trav. Angle Theta Ph. Alpha Ph. Beta Ph. t. Reward Eye Vert. Eye Horz.
E
1 Spike/s
95%CI
empirical spike-triggered average
p-gam coupled
p-gam uncoupled
time from 'sender' spike (seconds)

Figure S6. Coupling filters account for inter-neuron correlations. A. Example tuning, spike-history, and coupling filters estimated by the P-GAM. From top to bottom: Raw (gray) and P-GAM reconstructed (black) filters for task-variables (demonstrating the model's ability to account for spiking activity), spike history filters (showing the characteristic refractory period of single units), and coupling. **B.** Pseudo-R^2 for uncoupled (x-axis) and coupled (y-axis) models, showing that P-GAM encoding models allowing for neuron-to-neuron coupling accounted better for observed spike trains. Each dot is a neuron and the identity line in red (note different range in x and y). **C.** Five snippets (each 30 seconds long) of "observed" neural activity (convolved with a Gaussian filter with a standard deviation of 20ms, black), as well as predicted firing rates with a P-GAM allowing for coupling (coupled model, red), or not (uncoupled mode, blue). **D.** Correlation between tuning functions to different task variables (x-axis) estimated with the coupled and uncoupled model (y-axis = r-values, average r = 0.93, all variables above r = 0.85). The different brain areas are colored (MSTd = green; 7a = blue, dlPFC = red), and there was no difference in the stability of these tuning functions across brain area (one-way ANOVA, p = 0.68). Note, however, there was a difference in their gain-modulation, being most readily modulated in MSTd than 7a or dlPFC (see **Fig. 2B**). **E.** Example (12) across-units spike-triggered averages (i.e., average firing rate of "receiver" neuron condition on a spike from a "sender" unit), as empirically estimated (black) and estimated via coupled (red) or uncoupled (blue) P-GAM encoding models. Ten of these neurons (all but bottom row, 3rd and 4th from the left; transparent colors) were estimated as coupled by the P-GAM, while the last two (shown as a negative control) were determined not to be coupled. Of note, the coupled model (red) is able to recapitulate neuron-to-neuron dynamics, while the uncoupled model is not (mean R^2, coupled model, 0.65 ± 0.02; uncoupled model, R^2 = 0.26 ± 0.06; p = 0.0003). Further, we determine 95%CIs for each sender-receiver pair by shuffling spike times of the "sender" unit 1000 times and re-computing spike-triggered averages. On average, the P-GAM estimated 11.31% of unit pairs to be coupled, while this number was much higher (39.8%) according to permutation testing on raw data. This discrepancy highlights the critical need for an encoding model, accounting and explaining away signal-correlations. In other words, while the empirical spike-triggered averages may be driven by both neurons responding to a "signal", the model estimated pairs are conditioned on this other input, and thus results in a more conservative (i.e., statistically robust) estimate. When both the coupled model and empirically-estimated spike triggered averages agreed on indexing a coupled pair, their R^2 was 0.75. In conclusion, the real benefit to the coupled P-GAM is not in better accounting for spike times of a single neuron (**B** and **C**) but in being able to account for neuron-to-neuron dynamics above and beyond signal correlations (**E,** also see **Figure 4C** in Lakshminarasimhan et al., 2023). Source data are provided as a Source data file.

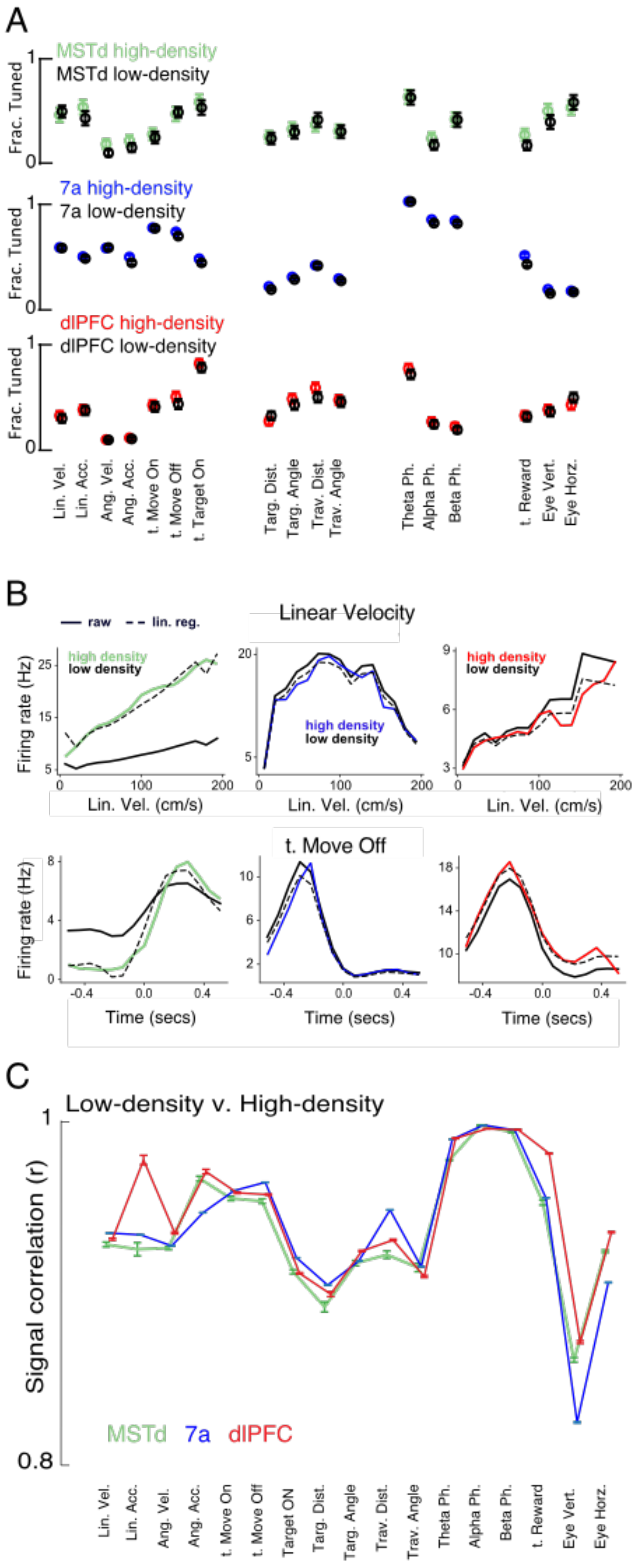
A
MSTd high-density
MSTd low-density
7a high-density
7a low-density
dlPFC high-density
dlPFC low-density
Frac. Tuned
Lin. Vel.
Lin. Acc.
Ang. Vel.
Ang. Acc.
t. Move On
t. Move Off
t. Target On
Targ. Dist.
Targ. Angle
Trav. Dist.
Trav. Angle
Theta Ph.
Alpha Ph.
Beta Ph.
t. Reward
Eye Vert.
Eye Horz.
B
raw
lin. reg.
Linear Velocity
high density
low density
Firing rate (Hz)
Lin. Vel. (cm/s)
t. Move Off
Time (secs)
C
Low-density v. High-density
Signal correlation (r)
MSTd 7a dlPFC
Target ON

Figure S7. Signal correlations in high and low optic-flow density conditions. A. Fraction of neurons tuned. High- (colored) and low-density (black) optic flow conditions did not result in a different fraction of neurons tuned to different task variables in MSTd (green), area 7a (blue), or dlPFC (red). Error bars are +/- 95%CI across neurons in all sessions. **B.** Example tuning functions to linear velocity (top) and time from movement offset (bottom) for each neural area (green = MSTd; blue = area 7a; red = dlPFC) during high- (colored) and low- (black) optic flow density conditions. In addition to the raw data (solid lines), we also demonstrate the re-scaling via linear regression from low- and high-density condition (dashed lines) in order to estimate gain modulations. The examples are representative, in that they demonstrate (1) a stability in the overall shape of tuning functions across all areas (see main text), and (2) no or weak gain modulation in 7a and dlPFC, but a strong gain modulation in MSTd (see **Fig. 2B**). **C.** Average correlation (r-values) between the tuning functions to different task variables (x-axis) estimated in high and low optic-flow density conditions. The tuning functions are very stable (grand mean r = 0.92) and not different across brain areas (MSTd = green; 7a = blue; dlPFC = red). Error bars are ± 1 S.E.M across neurons (all sessions combined). Source data are provided as a Source data file.

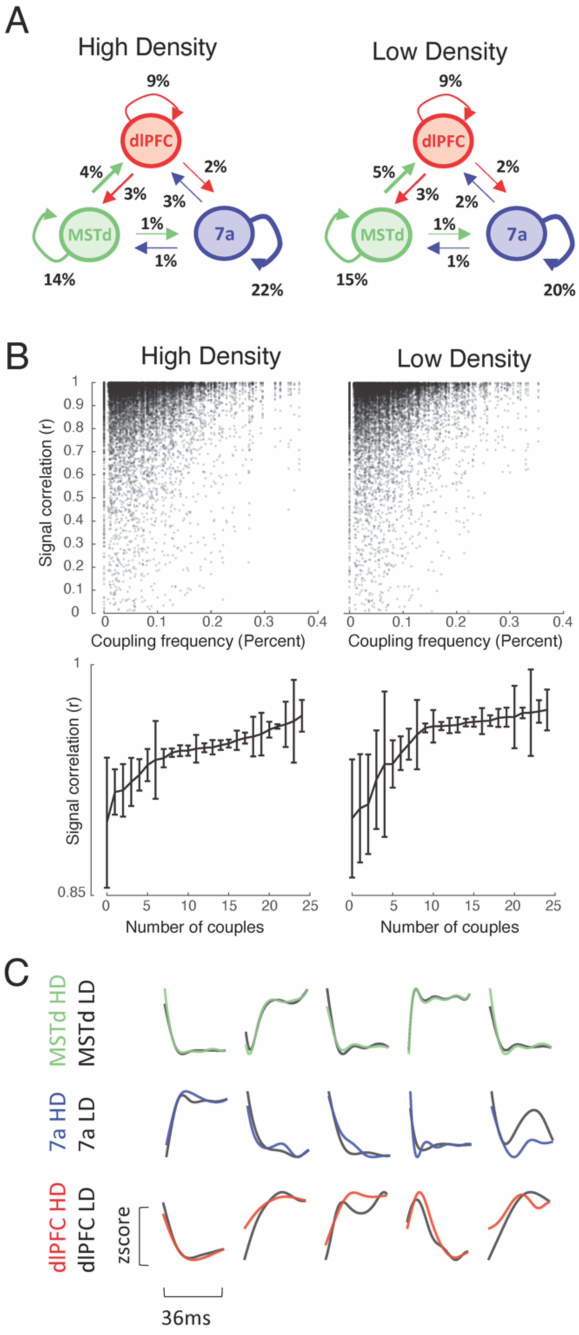

Figure S8. Noise correlations in high and low optic-flow density conditions. A. Fraction of units coupled within and across areas in high- (left) and low-density (right) conditions**.** Thickness of arrows, and inset percentage indicate the fraction of neurons coupled within and across areas. An arrow projecting from e.g., MSTd to dlPFC indicates that the firing of a neuron in MSTd will subsequently influence spiking activity in dlPFC. **B.** Signal correlation (i.e., r-value between a given tuning function estimated in low and high density) as a function of the fraction of simultaneously recorded neurons (within brain area) the unit is coupled to (top), or the total number of units (within and across area) the neuron is coupled to (regardless of the number of simultaneously recorded neurons, bottom). The units a given neuron is significantly coupled to may change across densities, and thus we perform the abovementioned computation when coupling frequency (top) and total number of coupled units (bottom) is computed either in the high (left) or low (right) optic flow density condition. In all cases, tuning stability is greater for neurons coupled to more other neurons. **C.** Coupling filters remain stable across optic flow densities in MSTd (green), but less so in area 7a (blue) and dlPFC (red). Five examples are shown for each area (high-density colored and low-density in black; shown in Kernel-space). Coupling functions within area had a length of 36ms (see[16] and *Methods* for further detail). See **Figure S6E** (red) for example coupling filters in Hz-space, and protracted in time (1.2 seconds). Source data are provided as a Source data file.

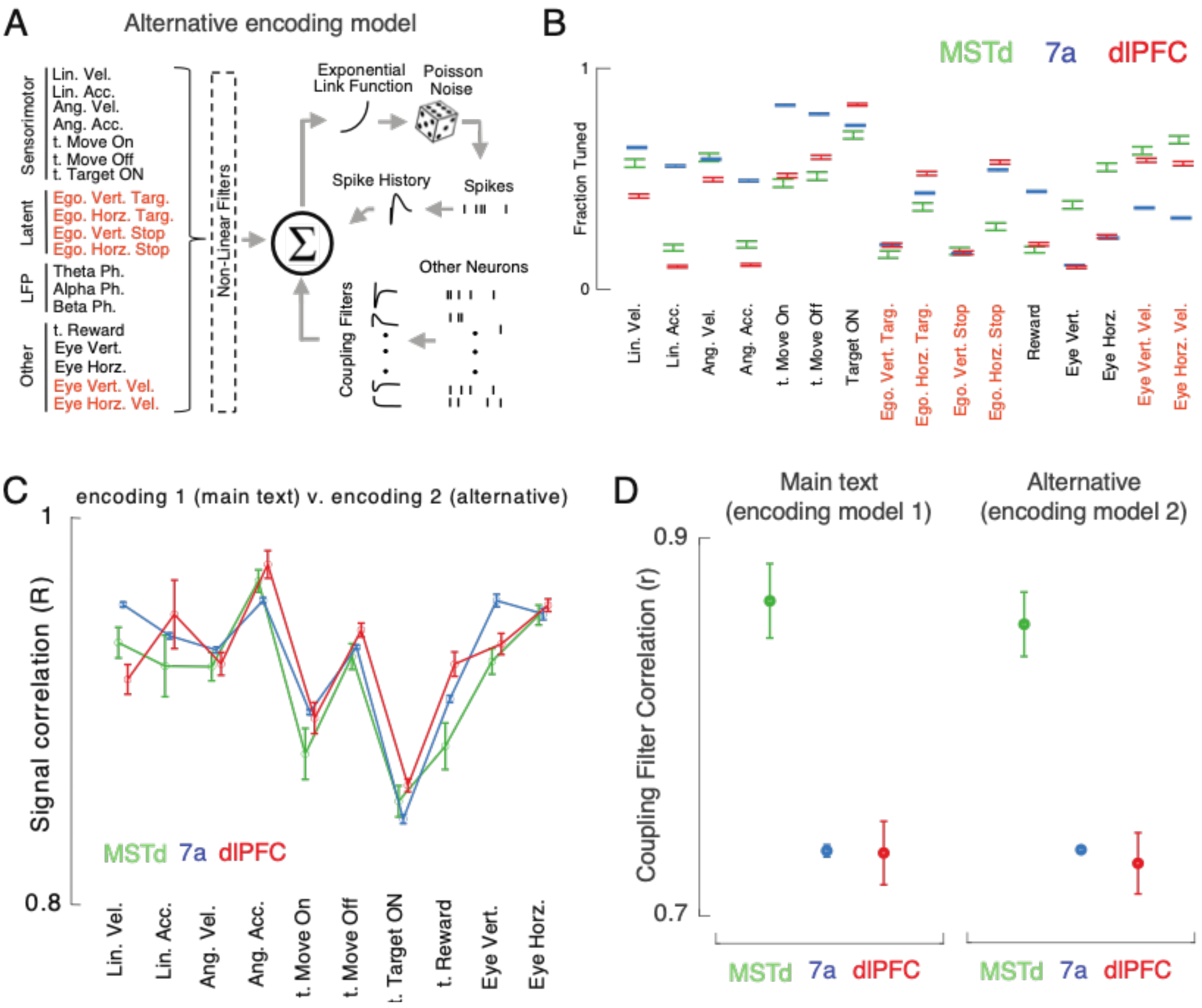

Figure S9. An alternative encoding model reproduces the dynamic remapping of noise correlations in 7a and dlPFC. A. Alternative encoding scheme (contrast with **Fig. 2A,** differences highlighted in orange here). This alternative P-GAM expressed distances to target in egocentric coordinates. Namely, instead of conceiving the targets in a three-dimensional space with depth, the targets are now projected onto the two-dimensional coordinates of a screen. Instead of a radial and angular distance to target, now the targets are expressed by a vertical and horizonal angle vis-à-vis the ongoing location of gaze (also on the screen). Further, we test the hypothesis that animals may not path integrate distances from origin, but from their eventual stopping locations, and thus we include egocentric distances (vertical and horizontal) to the eventual stopping location. Lastly, we include not only eye positions, but also eye velocity. Figure adapted from REF[35]. **B.** Fraction of neurons tuned to the different variables in the alternative encoding model. These are congruent with prior results (REF[35] and **Fig. S7A**), showing a patterned mixed selectivity, with greater coding for sensorimotor variables in 7a and of latent variables of dlPFC. Further, the results show greatest coding of eye velocity in MSTd. **C.** Signal correlations (r-value between tuning functions estimated in the different encoding models) are a function of brain area (MSTd in green, 7a in blue, and dlPFC in red) and task variable (restricted to the subset of variables present in both the main-text encoding model and the alternative one presented here). **D.** Coupling filter correlation (r-value in shape of coupling function in low- and high-density optic flow condition) as a function of brain area (MSTd in green, 7a in blue, and dlPFC in red) and encoding schema (main text on the left, and alternative P-GAM on the right). The findings replicate the results demonstrating greater remapping in 7a and dlPFC as opposed to MSTd with the change of optic-flow density. Error bars are ± 1 S.E.M. Source data are provided as a Source data file.

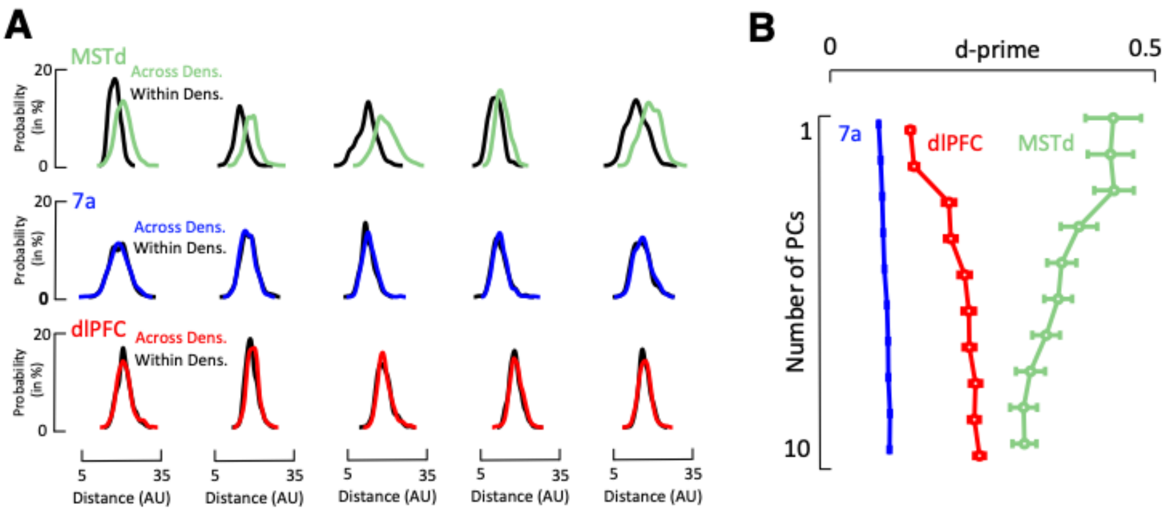

Figure S10. Population codes remain stable in 7a and dlPFC but not MSTd. A. Distribution of distances between high- and low-density conditions in latent space. Five example sessions are shown for MSTd (green), 7a (blue) and dlPFC (red). In each session, 1000 trials are paired based on their similarity in target location and steering behavior, either within (i.e., HD/HD or LD/LD) or across (i.e., HD/LD) density conditions. Each trial is then projected onto PC space (2D) and their distance is computed. The histograms show are the distances in latent space between those 1000 trials. **B. Summary statistics.** The distance between distributions (within vs. across, as in **A**) is computed by d-prime. Latent neural trajectories are more dissimilar across density conditions in MSTd (green) than 7a (blue) or dlPFC (red) regardless of the number of PCs used. As we index higher-order PCs, we observe that dlPFC become different between high- and low-density conditions. Area 7a is always stable. Error bars are ± 1 S.E.M. Source data are provided as a Source data file.

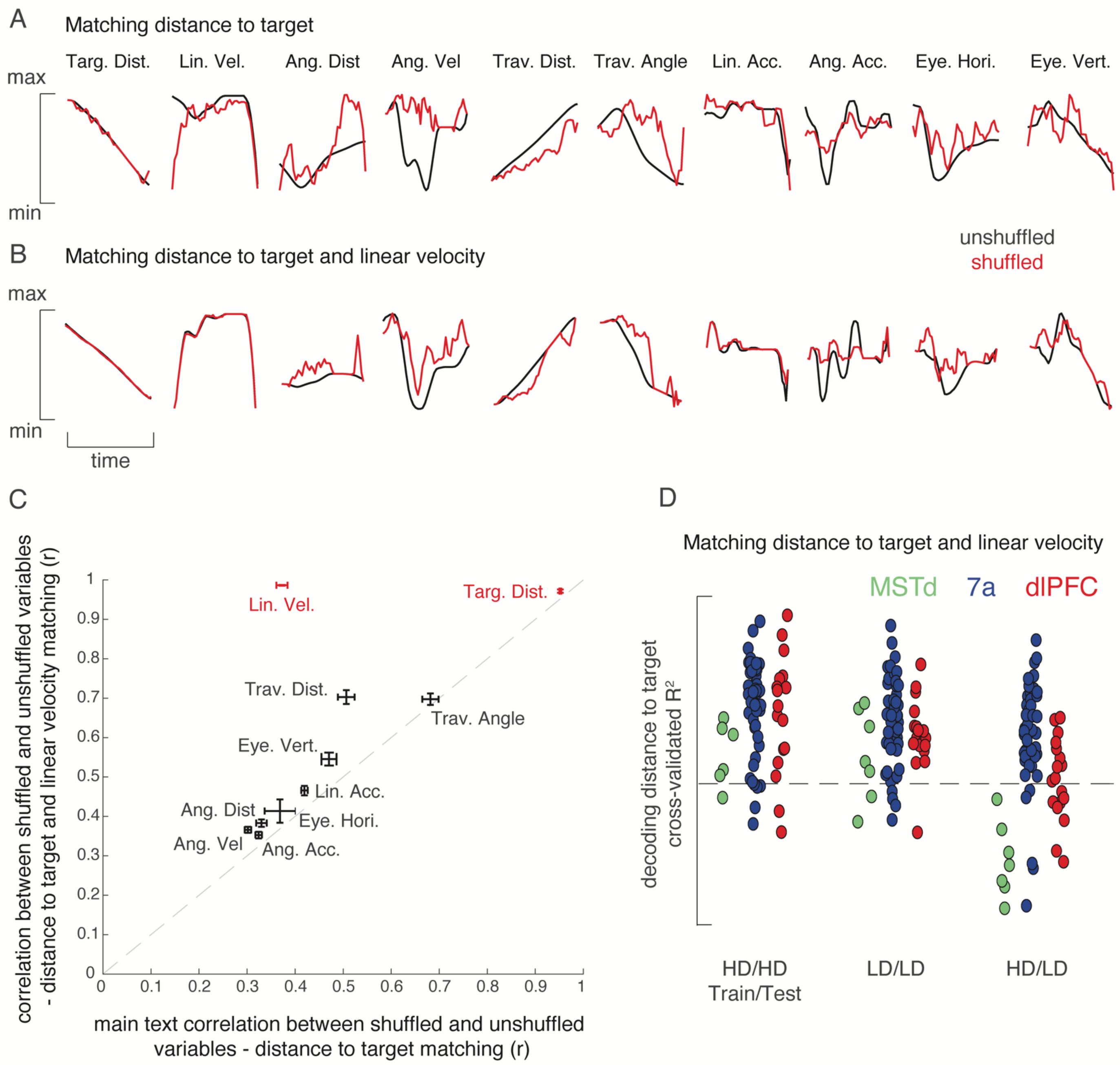

Figure S11. Artificially removing noise correlations. To test for the impact of noise correlations in allowing for distance to target cross-decoding, we match time epochs with the same distance to target (and thus output from the tuning function) and shuffle neural activity like-for-like. Meaning, we conserve signal correlations while eliminating the naturally occurring noise correlations. **A.** Examples showing "unshuffled" (black) and shuffled (red) task variables, when matching only distance to target. The correspondence between unshuffled and shuffled distance to target is very high by construction. The rest of variables (e.g., linear velocity, eye positions) are moderately correlated given the correlation between variables in this naturalistic task (grand average r ~ 0.15). **B.** For a more stringent test (vs. main text) here we additionally match linear velocity. Now distance to target and linear velocity are very well matched, by construction. The rest of variables also become slightly better correlated. **C.** Scatter plot (error bars are ± 1 S.E.M.) showing the average correlation between task variables in shuffled and unshuffled conditions, when matching solely distance to target (x-axis), or both distance to target and linear velocity (y-axis). In the latter case, all task variables are correlated in shuffled and unshuffled conditions above r = 0.3. **D.** Decoding of distance to target (CV R^2, y-axis) in high-density (HD/HD), low-density (LD/LD), or across contexts (HD/LD) when eliminating noise correlations and matching for both distance to target and linear velocity. As in the main text (distance to target matching), across context decoding was abolished by the lack of noise correlations in dlPFC (red) but not in 7a

(blue). MSTd (green) cannot cross-decode even with noise correlations (see main text). Source data are provided as a Source data file.

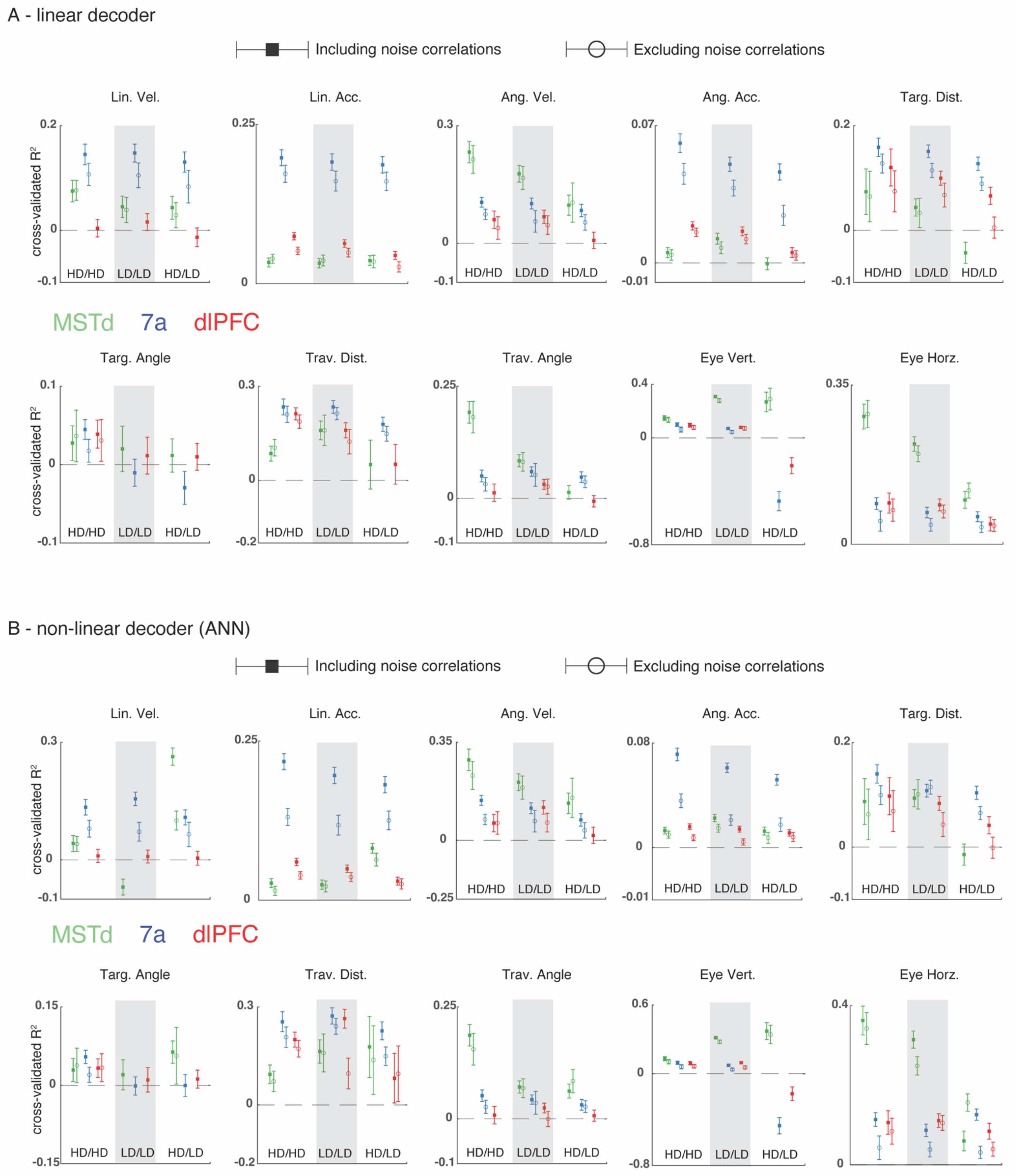

Figure S12. Decoding task variables within and across contexts, including and excluding noise correlations. A. Decoding cross-validated R^2 for each continuous task variable (panels) and brain area (MSTd = green, 7a = blue, dlPFC = red), both within contexts (HD/HD, LD/LD) and across contexts (HD/LD). Squared filled markers are used to indicate decoding including noise correlations (i.e., "unshuffled") while empty circles are used to indicate decoding excluding noise correlations. The latter are only plotted when the decoding for a specific area/task variable is significant when including task variables. Error bars are ± 95%CI, and thus non-overlapping error bars with CV = 0 (dashed line) is significant. The only brain area/task variable that is decodable across context with noise correlations but not without is distance to target in

dlPFC. **B.** Same as **A**, while employing a non-linear artificial neural network for decoding. Results are replicated. Source data are provided as a Source data file.

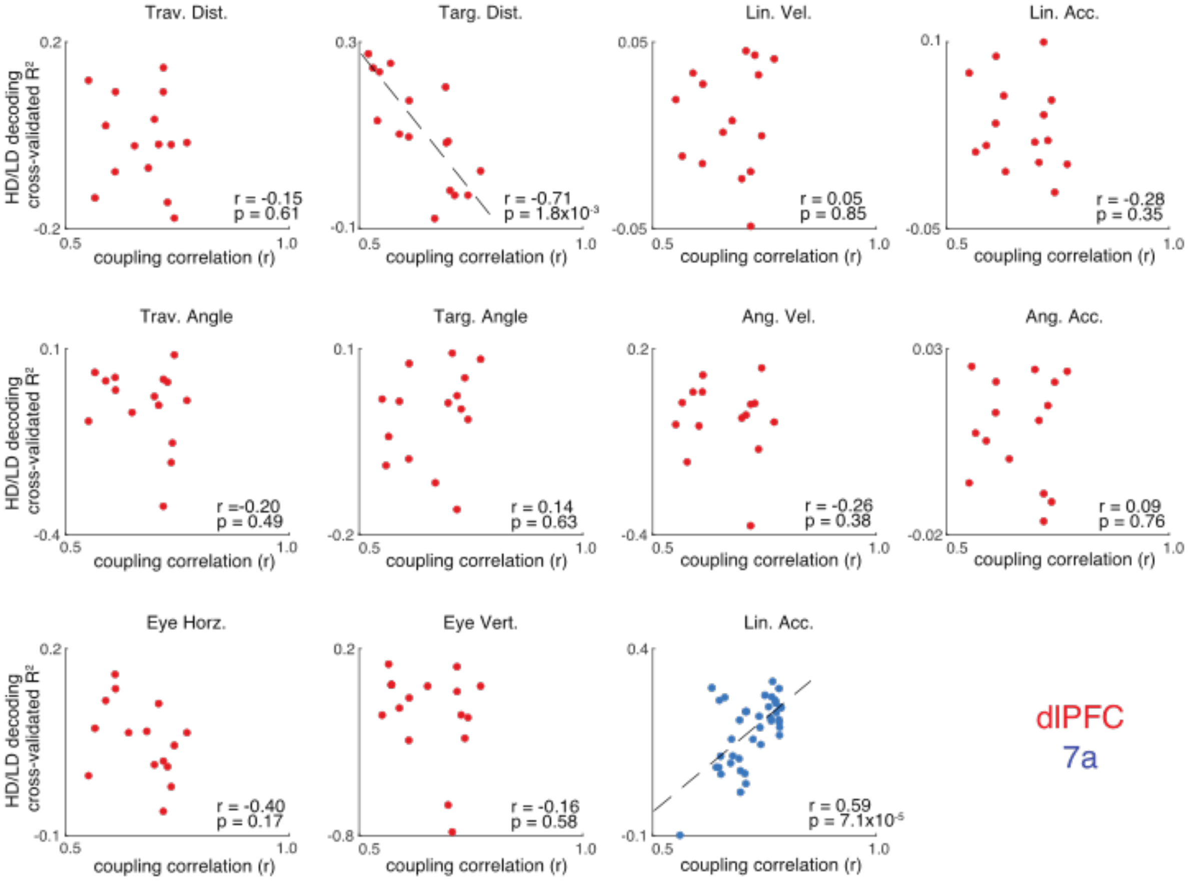

Figure S13. Relations between cross-context decoding and coupling filter stability. All continuous variables are showing for dlPFC (red). The only significant correlation is between the stability to coupling filters (within a session), and the ability of that session for cross-context decoding of distance to target (top row, second column). The only significant relationship for area 7a is between coupling stability and linear acceleration (bottom row, third column). Source data are provided as a Source data file.

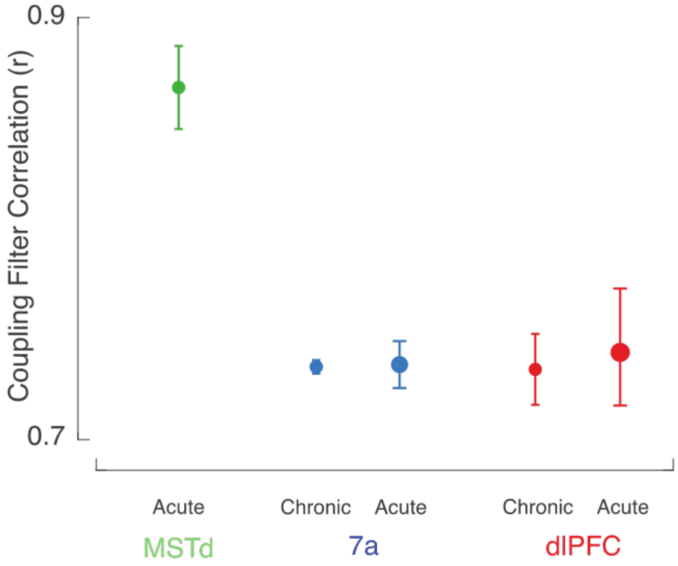

Figure S14. Coupling filter correlation as a function of probe type. MSTd recordings were undertaken exclusively with acute recordings, due to its anatomical location. Recordings in 7a and dlPFC were undertaken both with acute and chronic preparations. To examine if probe type impacted the results (and control for the possibility that units recorded on different days with chronic preparations are not independent), we examined coupling function correlations as a function of probe type. Even when restricting our analyses to acute recordings, noise correlations in 7a and dlPFC more readily remapped as a function of optic-flow context (Kruskal-Wallis, $p = 0.0035$). There was no difference in coupling filter correlation as a function of recording technique in 7a ($p = 0.71$) nor dlPFC ($p = 0.79$). Source data are provided as a Source data file.
